# Consistent vehicle trajectory extraction from aerial recordings using oriented object detection

**DOI:** 10.1038/s41598-025-12301-2

**Published:** 2025-07-28

**Authors:** Kevin Riehl, Shaimaa K. El-Baklish, Anastasios Kouvelas, Michail A. Makridis

**Affiliations:** Traffic Engineering Group, Institute for Transport Planning and Systems, ETH Zurich, Stefano-Franscini-Platz 5, 8093 Zurich, Switzerland

**Keywords:** Civil engineering, Engineering, Electrical and electronic engineering

## Abstract

Vehicle trajectories offer valuable insights for a wide range of road transportation applications. Due to the rise of drone technology, a growing branch of literature explores optical vehicle trajectory extraction from aerial videos, where object detection using neural networks is an important component. Horizontal bounding box object detection struggles to differentiate well between rotated vehicles, especially when dealing with complex backgrounds or densely packed vehicles. This work proposes a generalizable computation pipeline that leverages angular information to extract high-quality trajectories starting from video recordings and ending in trajectories in Cartesian and lane coordinates. A trajectory reconstruction algorithm is designed to be vehicle- and driver-informed and to maximize the physical consistency of the reconstructed trajectories both on the individual vehicles’ and platoon levels. A comprehensive benchmark of 18 object detection models on a real-world video dataset demonstrates how oriented object detection and the use of angular information can be used to significantly improve the consistency of extracted trajectories (15% better internal, and 20% better platoon consistency), and that orientation-informed trajectories can be reconstructed to lane coordinates of higher quality. The reconstructed vehicle trajectories better capture car-following and traffic dynamics, thereby improving their usability for traffic flow studies.

## Introduction

Vehicle trajectories offer valuable insights for a wide range of road transportation applications and research fields. Trajectories allow to model realistic driving behaviours, such as car-following & lane-changing, and real-world traffic patterns, such as oscillation propagation & capacity drops. In addition to that, trajectories are helpful when developing driving safety systems, analysing energy consumption of vehicles, and support road infrastructure planning, design and control^1–3^.

The recording of trajectories can involve probe vehicles with GPS sensors, or optical trajectory extraction with cameras. Optical trajectory extraction is more precise due to the higher resolution of video streams in time and space, cheaper, less prone to sensor synchronization issues, and allow to cover all vehicles on a road in the field of view of a camera. Video material for optical vehicle trajectory extraction originates from stationary, traffic camera footage (e.g., installed, fix highway and city cameras), online video sources (e.g., YouTube), and aerial videos from Unmanned Aerial Vehicles (UAVs)^4^.

The detection of vehicles using object detection is an important component of optical trajectory extraction systems. For a given image, object detection algorithms return a list of identified objects, including their coordinates on the image, and a probability (confidence) of correctly identifying an object. The coordinates on the image are described as a rectangular, horizontal bounding box (HBB), covering the detected object. When faced with objects of arbitrary size, scale, or orientation, the HBBs often contain complex backgrounds around the object of interest which affects detection performance. As a consequence, the detection of vehicles can be challenging due to issues such as occlusion, dynamic backgrounds, and changing vehicle appearance under different light and weather conditions^5^.

Contrary to conventional object detection, oriented object detection provides oriented bounding boxes (OBB), that more precisely enclose the objects of interest, decrease noise of backgrounds, and reduce object occlusion in images^6,7^. To not only know the position, but also the orientation of vehicles could substantially improve the estimation of vehicle trajectories from video streams, driver safety assessment, and the modelling of driver behaviour^8^. However, previous works in the field of vehicle trajectory extraction overlooked oriented object detection. Therefore, this work sets out to explore how oriented object detection can enhance the quality of extracted vehicle trajectories from aerial video streams, and benchmarks the performance of state-of-the-art OBB models.

In this work, we propose a computation pipeline for optical vehicle trajectory extraction and trajectory reconstruction, that leverages Hungarian matching, and Rauch-Tung-Striebel, extended Kalman Filtering. Moreover, we benchmark 18 object detection models, and process more than 40 minutes, high definition video footage from an aerial perspective, in which 14 vehicles drive curvy trajectories on a circular road at a Swiss driver training centre^9^. The results indicate that employment of OBB annotations and angular information can significantly improve the quality of trajectories, with more than 15% increased internal and 20% increased platoon consistency. Trajectory reconstruction results in a similar assessment.

The contribution of this work is to demonstrate the benefits of oriented object detection in the context optical vehicle trajectory extraction, by providing an exhaustive benchmark of state-of-the-art object detection models. To the best of our knowledge, such a comprehensive benchmark is unique and missing in such a form to date in the literature. Moreover, this work offers a robust and physically consistent vehicle trajectory extraction pipeline, as an open source software repository. It can be found on GitHub: https://github.com/DerKevinRiehl/trajectory_analysis. Finally, this work not only develops and evaluates a trajectory extraction method according to conventional consistency measures, but in addition to that offers an application-specific, downstream analysis in the context trajectory reconstruction and vehicle dynamics analysis.

## Related works

Previous works on optical vehicle trajectory extraction focused on cars on highways and urban intersections; however studies also explored automated container vehicles at ports^10^, motorcycles, and mixed vehicle traffic^11^. Besides the trajectory studies aimed for accurate vehicle speed estimation, intersection queue length estimation, vehicle classification, traffic volumes measurement, and the accident risk levels identification at sections of the road^10,12–15^.

The process of optical vehicle trajectory extraction usually consists of three steps: (i) vehicle detection, (ii) homography, and (iii) trajectory connection. Object detection refers to annotating image regions that relate to object instances. For this step, works employ techniques from classic computer vision for object detection, including edge detection, key point extraction, feature motion analysis, and morphologic operations^4,16,17^. The majority of works however, uses deep learning techniques based on convolutional neural network (CNN) architectures, including *Yolo*, *ResNet*, *Inception, R-CNN*, *Grid R-CNN*, *Faster R-CNN*^10^. Homography refers to coordinate transformation of positions on recorded images (camera coordinates) to real world coordinates such as Cartesian, Frenet, and lane coordinate systems^18^. Trajectory connection refers to matching single positions that relate to the trajectory of one specific car, and fill gaps in time series of vehicle trajectories^11^. This step employs matching algorithms (e.g., Hungarian algorithm), and signal processing for denoising (e.g., Kalman Filter, Wavelet transform)^10^. Some works also employ *DeepSORT*^19^, where the Hungarian matching is based on a pre-trained CNN acting as an appearance descriptor and the Mahalanobis distance between Kalman-predicted object motion and measurements from the detection pipeline.

Various experiments using video recordings from an aerial view, resulted in rich vehicle trajectory datasets, including *pNEUMA* for the city of Athens^20^, *VisDrone2019* for various modalities in an urban context of Tianjin in China^21^, *UAVDT* for various vehicle types, road and weather conditions^22^, *highD* for German highways^23^, *CitySim* for various intersection types in the United States^8^, *NGSIM* & *HIGH-SIM* for various motorways in the United States^4,24^, and *SIND* for more than 15 intersections in China^25^. The direction of vehicles is solely provided by *CitySim* dataset, and considered as a valuable, additional information when analysing driver behaviour and assessing road safety^8^ (the direction of vehicles is estimated based on their trajectories, but not from the images themselves).

Vehicle detection on aerial images can be challenging. A previous assessment on vehicle detection to benchmark HBB-based *Yolov8* models was carried out in^26,27^. Challenges occur due to camera motion (shaking), low image resolution, small vehicle sizes at too high distances, densely packed objects, and the occlusion of objects and their HBB bounding boxes^11^. The detection performance can be improved using oriented object detection, as OBB bounding boxes obtain an improved detection accuracy, better fit-to-shape, reduced output ambiguity, less overlap and fewer over-counting^28^.

Oriented object detection models can be based on existing deep learning models for object detection^6^, on segmentation-based-approaches^29^, butterfly approaches^30^, and circular Gaussian distribution approaches^31^. OBB datasets to train and evaluate these models include *DOTA*^32^, *iSAID*^33^ and *HRSC2016*^34^.

## Methods

**Figure 1:**
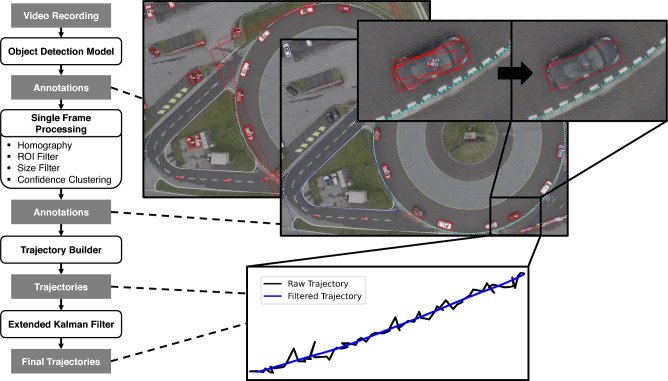
Vehicle trajectory extraction pipeline. The proposed vehicle trajectory extraction pipeline starts at video material. Using an object detection model, raw annotations are generated. These raw annotations are filtered based on region of interest and size, transformed to Cartesian coordinates, and clustered. The filtered annotations are then matched to vehicle trajectories. Finally, the vehicle trajectories are filtered with an Extended Kalman Filter to derive the final vehicle trajectories.

We propose a pipeline to derive trajectories from single frame vehicle annotations using OBB and HBB models. The pipeline consists of three steps and is summarized in Fig. 1. In the first step, the annotations of a single frame are processed, filtered, and includes the transformation from image to real world coordinates (homography). In the second step, trajectories are derived by matching annotations on a frame-to-frame basis. In the third step, gaps in a single vehicle trajectory are filled and processed using an Extended Kalman Filter. The Extended Kalman Filter can optionally be orientation-informed, which enables significantly more consistent trajectories. As a result, vehicle trajectories in Cartesian coordinates are produced. Furthermore, a vehicle trajectory reconstruction algorithm, translates these trajectories into lane coordinates and provides physically-plausible velocity and acceleration information.

### Object detection models

For vehicle detection we employ two groups of model, OBB and HBB object detection models, which are outlined in Table 1. The models were all (re)trained on DOTA^32^, a dedicated dataset including aerial images and vehicle annotations. DOTA offers OBB annotations only; hence, annotations were transformed in order to train the HBB models. The HBB annotations were derived from the OBB annotations by using the tightest HBB to enclose the OBB. The center of the annotations (vehicle position) is not changed due to this transformation.. The selection of models allows a direct comparison between OBB and HBB detection.

**Table 1:** Object detection models.

$\hbox {N}^{\,circ}$	Type	Family	Neural Network Architecture	Ref.	#Params(M)	mAP(50-95)	(50)
1	OBB	openmmlab	cfa_r50_fpn_40e_dota_oc	^35^	73.5	46.27	73.45
2	OBB	openmmlab	oriented_rcnn_r50_fpn_1x_dota_le90	^36^	82.6	47.68	75.69
3	OBB	openmmlab	redet_re50_refpn_1x_dota_ms_rr_le90	^37^	65.3	50.32	79.87
4	OBB	openmmlab	roi_trans_r50_fpn_1x_dota_ms_le90	^38^	110.5	50.19	79.66
5	OBB	openmmlab	rotated_retinanet_obb_r50_fpn_1x_dota_ms_rr_le90	^39^	73.2	48.20	76.50
6	OBB	openmmlab	s2anet_r50_fpn_fp16_1x_dota_le135	^40^	77.5	46.74	74.19
7	OBB	ultralytics	yolov8n-obb	^41^	3.1	49.15	78.01
8	OBB	ultralytics	yolov8s-obb	^41^	11.4	50.10	79.52
9	OBB	ultralytics	yolov8m-obb	^41^	26.4	50.75	80.55
10	OBB	ultralytics	yolov8l-obb	^41^	44.5	50.86	80.73
11	OBB	ultralytics	yolov8x-obb	^41^	69.5	51.26	81.36
12	HBB	openmmlab	faster-rcnn_r50_fpn_ms-3x_coco$*$	^42^	41.4	26.10	39.80
13	HBB	openmmlab	retinanet_r50_fpn_ms-640-800-3x_coco$*$	^39^	36.6	22.60	36.21
14	HBB	ultralytics	yolov8n$*$	^41^	3.2	27.63	44.35
15	HBB	ultralytics	yolov8s$*$	^41^	11.2	30.70	48.41
16	HBB	ultralytics	yolov8m$*$	^41^	25.9	32.94	51.05
17	HBB	ultralytics	yolov8l$*$	^41^	43.7	33.62	51.74
18	HBB	ultralytics	yolov8x$*$	^41^	68.2	35.55	53.86

* denotes the models which were retrained on the transformed *DOTA* dataset

### Single frame processing

First, we transform the image coordinates (in pixel) to real-world, Cartesian coordinates (in m), using a homography-transform of a characteristic object in the image. In our case, we have a characteristic, large circular border of the circular road. For each picture of the video, we extract location and size of the circle using Hough transform^43^. This allows for determining the distance of each vehicle relative to the circle center, and to calculate vehicle dimensions relative to the (known) diameter of the circle (65.17m). Afterwards, we filter all annotations based on whether they lie in a defined region of interest (in our case the circular road and small bay road), and based on vehicle-typical dimensions (widths 1.8-8.0m, heights 0.4-8.0m). Last but not least, it often occurred that object detection models provided multiple annotations for the same car, with slight variations of exact position, shape and orientation (in case of OBB models). Therefore, we cluster annotations that are close together (at most 3.0m distance), and determine the annotation with the highest confidence probability (as stated by the object detection model). To summarize, this step requires following parameters: (i) transformation function from image to Cartesian coordinates, (ii) pre-defined region of interest, (iii) accepted vehicle dimensions.

### Trajectory builder

We build vehicle trajectories based on the matched annotations of two consecutive images of the video stream. First, we define vehicle labels to each vehicle in the first image of the video stream. Next, we apply Hungarian matching^44^, to match annotations of the next image with annotations of the previous image. We consider a maximum allowed distance of a vehicle between two images of 2m, as even though our video’s temporal resolution is 25 frames per seconds, there can be gaps in annotation over multiple images, depending on the quality of annotations. Annotations that finally cannot be matched to the trajectory of a specific vehicle are discarded. To summarize, this step requires following parameters: (i) initial annotation of all relevant vehicles, (ii) accepted maximum matching-distance.

### Extended Kalman filter

The trajectories from the previous step are a very good starting point, but suffer from noise and gaps in time series. To this end, vehicle trajectory reconstruction is needed. Multiple approaches exist in the literature, such as physics-informed approaches^45,46^ and optimization-based approaches^47^.

We apply a conditional, time-discrete, non-linear, Extended Kalman Filter^48,49^ to complete gaps in the trajectories and improve trajectory quality overall. The Kalman Filter employs a model that allows to describe and predict the behaviour of a system, and consider both model-based predictions and measurements during the filtering process. There are three steps involved: (i) an inference step (in case gaps are too large), (ii) a prediction step (which is performed at any point in time), and (iii) a correction step (when measurement data is available).

The system model consists of the following five (latent) vehicle states: (i) position *x*, (ii) position *y*, (iii) orientation $\alpha$, (iv) speed *v*, and angular velocity $\omega$. We denote the system state $X = \left[ x, y, \alpha , v, \omega \right]$. The system model relates the system states with each other in following form, where the state *X*[*k*] represents the state at discrete time *k* and $\Delta t$ represents the time between $k+1$ and *k*:

1 $$\begin{aligned} X[k+1] = f(X[k]) = \begin{pmatrix} x + cos(\alpha )v\Delta t \\ y + sin(\alpha )v\Delta t \\ \alpha + \omega \Delta t \\ v \\ \omega \end{pmatrix} \end{aligned}$$

Besides, there is an observable state *Y* that can be measured with some errors $\tilde{Y}$, and can be estimated by the system model $\hat{Y}$. We consider all system states to be observable. In case of HBB annotations, positional information is solely used to estimate $\tilde{Y}$. In case of OBB annotations, $\alpha$ and $\omega$ are derived from the orientation provided by the neural network The angles provided by the neural networks $\alpha$ need to be preprocessed. The angles of different models follow different orientations, and therefore need to be translated to an unified orientation. Moreover, the angles need to be bounded between 0 and 360 degrees (we do not allow for negative angles).. The additional angular information provided by OBB models is instrumental to generate better trajectories. The system model can be used to predict the observable state as follows:

2 $$\begin{aligned} \hat{Y}[k] = h(X[k]) \end{aligned}$$

During each step of the Kalman Filtering, the predicted and corrected state covariance matrices, $P_p$ and $P_c$ respectively, are estimated and updated, using the process and measurement noise covariance matrices, *Q* and *R* respectively. These covariance matrices need to be set initially and represent how trustworthy specific latent and observable states are, with higher values in *Q* and *R* representing less trust in estimates and measurements. The Extended Kalman Filter predicts the final trajectory $Y_K$ as described in Algorithm 1. In case the gaps in time series are larger than $g_{max}=25$ consecutive frames, the Extended Kalman Filter is replaced by Algorithm 2, where *t* is the last frame before the gap, and *G* is the gap length. A Nelder-Mead-Optimizer^50^ is used to estimate optimal *v* and $\omega$ of the system state before the gap, to reach the first available measurement after the gap as close as possible. The final trajectory is calculated as weighted average of a time-forward and time-backward Kalman-filtered trajectory, with weights depending on the distance to the previous available measurement, similar to a Rauch-Tung-Striebel fixed-interval smoothing^51^. For simplicity, we use the unit matrix for the error covariance matrices $Q = R = I$. The initialization of states $X_c[0]$ and $X_p[0]$ is determined as the mean from the first 30 points of the time series (in the context of this study’s frame rate). The initialization of state errors $P_c[0]$ and $P_p[0]$ is calculated accordingly as difference from the initial states. To summarize, this step requires following parameters: initialization of states $X_c[0]$, $X_p[0]$, initialization of errors $P_c[0]$, $P_p[0]$, error covariance matrices *Q*, *R*, and maximum gap length $g_{max}$.

**Algorithm 1:**
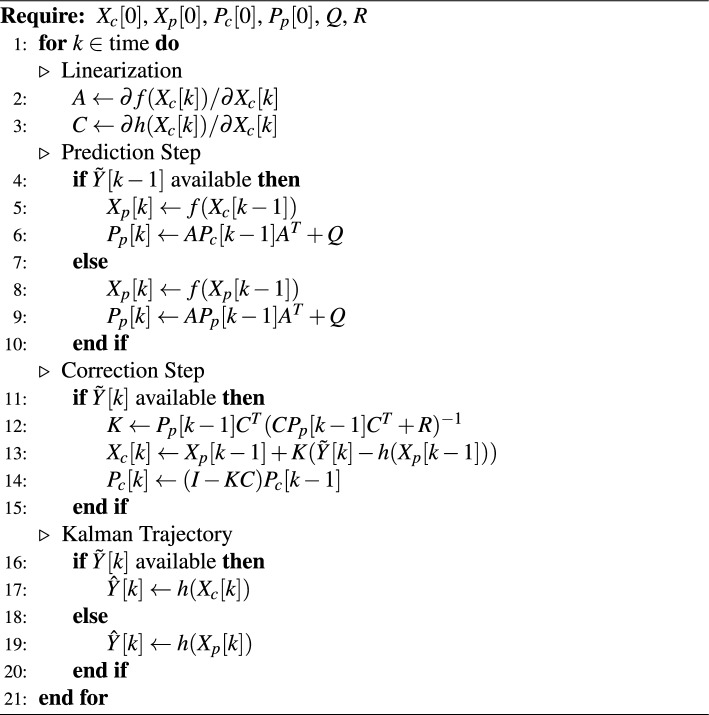
**Extended Kalman Filtering**

**Algorithm 2:**
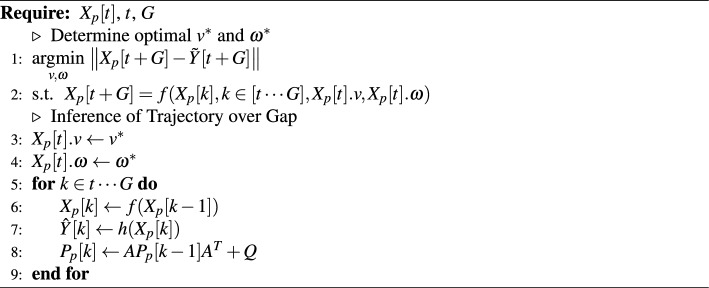
**Trajectory Gap Inference Filter**

### Evaluation measures

In order to evaluate the first stage of the proposed pipeline, we compare the quality of annotations before and after single frame processing. Due to our prior knowledge of 14 vehicles being on track, we asses the average number of annotations per image (frame). As we cluster vehicles based on annotation confidence, we assess the average confidence per annotation (Neural Network output).

To evaluate the second stage of the pipeline, we determine the completeness of vehicle trajectories after the trajectory building. Gaps in the trajectory time series arise due to consecutive frames where the vehicle was not annotated by the object detection model. We assess the share of all frames for which the vehicle trajectories could be identified, and analyse the gap length distribution. Moreover, we assess the plausibility of the unfiltered vehicle trajectories, by computing the standard deviation of vehicle width and heights, as well positional and angular noise measured in frame-to-frame-differences standard deviation.

To evaluate the third stage of the pipeline, we measure the quality of Kalman-filtered trajectories using OBB and HBB annotations using internal and platoon consistency based on travelled distance, as suggested in^4,24^, which requires translation of vehicles’ Cartesian coordinates to lane coordinates. These consistency measures reflect the bias in the extracted trajectories with respect to physically-plausible trajectories which should yield consistent speeds/accelerations. The internal consistency is expressed as follows

3 $$\begin{aligned} \varepsilon ^{S} = \hat{s}^n_t - \Big ( \hat{s}^n_0 + \int _0^t \hat{v}^n_\tau d\tau \Big ) \end{aligned}$$

where the $\hat{s}_t$ and $\hat{v}_t$ are the observed position and speed of a single vehicle *n*. Analogously, the platoon consistency is obtained for vehicle pairs (*n*, *m*),

4 $$\begin{aligned} \varepsilon ^{PS} = \Big ( \hat{s}^n_t - \hat{s}^m_t \Big ) - \Big ( \hat{s}^n_0 - \hat{s}^m_0 + \int _0^t \hat{v}^n_\tau - \hat{v}^m_\tau d\tau \Big ) \end{aligned}$$

where vehicle *n* is the subject vehicle and vehicle *m* is its following one.

### Trajectory reconstruction

Most vehicle trajectory datasets are published in Cartesian coordinates. However, downstream analysis of these vehicle trajectories (e.g., lane changing, car following, string stability) often requires transformation into lane coordinates. Lane coordinates allow for a simplified trajectory analysis that is aligned with the road geometry, and enable a comparison across different road segments. Moreover, many analyses not only require position of cars over time, but also additional state information such as velocity and acceleration.

**Figure 2:**
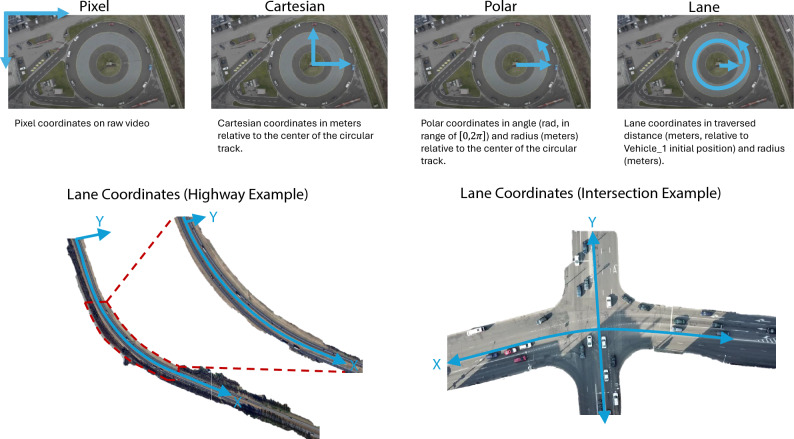
Coordinate systems used for the conducted car-following experiment and further examples.

The vehicles’ trajectories were expressed in Cartesian coordinates after the Kalman filtering. Through knowledge of the road geometry (i.e. circular track), we transformed the vehicles’ trajectories from Cartesian to polar coordinates, then to lane coordinates where the *x*-axis is aligned with the circular track. An isotonic regression is performed to ensure that the lane-coordinate vehicles’ positions are monotonically increasing. Figure 2 illustrates these coordinate systems for the conducted experiment. Furthermore, we present two examples where the lane coordinate axes are suggested for the proposed pipeline to be applicable. These suggested lane coordinate axes could be defined using splines to align with the curvilinear roadway.

**Figure 3:**
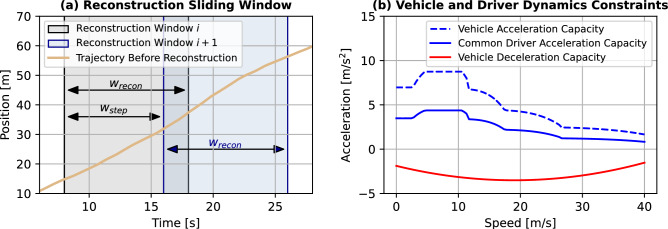
Illustration of the reconstruction sliding window and the vehicle and driver dynamics constraints expressed through the speed-acceleration curve to specify vehicle capabilities.

Nevertheless, trajectory extraction is still prone to measurement errors in terms of positional data; thus, corrupting the physical consistency and plausibility of the trajectory data. The smallest order of noise in positional data can even induce physically implausible speed and/or acceleration values in terms of vehicle and/or driver dynamics. For this purpose, we developed a trajectory reconstruction algorithm based on a constrained optimization. The reconstruction is conducted using a sliding window approach; where the reconstruction window and the sliding window step size are denoted by $w_{\text {recon}}$ and $w_{\text {step}}$ (in discrete time steps) respectively. For overlapping reconstruction windows, one needs to set $w_{\text {step}} \le w_{\text {recon}}$. Please refer to Fig. 3a for a visualization of such a sliding window.

Within the reconstruction window, we define the following variables.

5a $$\begin{aligned}&X_{\text {recon}} = \begin{bmatrix} x_{\text {recon}}(k)&\dots&x_{\text {recon}}(k+w_{\text {recon}}+1) \end{bmatrix}^T \end{aligned}$$

5b $$\begin{aligned}&V_{\text {recon}} = \begin{bmatrix} v_{\text {recon}}(k)&\dots&v_{\text {recon}}(k+w_{\text {recon}}) \end{bmatrix}^T, \qquad v_{\text {recon}}(k) = \frac{x_{\text {recon}}(k+1)-x_{\text {recon}}(k)}{\Delta t} \end{aligned}$$

5c $$\begin{aligned}&A_{\text {recon}} = \begin{bmatrix} a_{\text {recon}}(k)&\dots&a_{\text {recon}}(k+w_{\text {recon}}-1) \end{bmatrix}^T, \qquad a_{\text {recon}}(k) = \frac{v_{\text {recon}}(k+1)-v_{\text {recon}}(k)}{\Delta t} \end{aligned}$$

Here, $\Delta t$ denotes the sampling time interval and *k* is the discrete time step. The reconstructed trajectory variables are $x_{\text {recon}}$, $v_{\text {recon}}$ and $a_{\text {recon}}$ for the position, speed, and acceleration of an ego vehicle in lane coordinates respectively.

The constrained optimization problem for the reconstruction is now formulated as follows.

6a $$\begin{aligned} V_{\text {recon}}^\star = \arg&\min _{V_{\text {recon}}} \frac{\lambda _N}{w_{\text {recon}}+1}\left\Vert V_{\text {recon}} - V_{\text {filt}} \right\Vert _2^2 + \frac{\lambda _A}{w_{\text {recon}}} \left\Vert A_{\text {recon}} - \mu _{A_{\text {recon}}} \right\Vert _2^2 + \lambda _{DC} \sum _{\tau =k}^{k+w_{\text {recon}}-1} \frac{a_{\text {recon}}(\tau )}{d a_{\text {max}}}-1 \end{aligned}$$

6b $$\begin{aligned} \text {s.t. }&V_{\text {recon}} \ge 0 \end{aligned}$$

6c $$\begin{aligned}&d_{\text {min}} \le A_{\text {recon}} \le a_{\text {max}} \end{aligned}$$

6d $$\begin{aligned}&x(k+w_{\text {recon}}+1) - x(k) = \Delta t \sum _{\tau =k}^{k+w_{\text {recon}}} v_{\text {recon}}(\tau ) \end{aligned}$$

6e $$\begin{aligned}&x_{\text {prec}}(\eta +1) - L_{\text {prec}} - \Big ( x(k) + \Delta t \sum _{\tau =k}^{\eta } v_{\text {recon}}(\tau ) \Big ) \ge s_{\text {min}}, \quad \forall \eta \in \{ k, \dots k+w_{\text {recon}} \} \end{aligned}$$

This optimization problem is formulated with three objectives in mind — noise reduction, satisfaction of vehicle dynamics constraints, and compliance with common driver dynamics. The vehicle dynamics constraints are defined via the MFC microsimulation model^52,53^. The MFC model defines the maximum acceleration capacity $a_p(v)$ and the minimum comfortable deceleration capacity $d_p(v)$ of a specific vehicle at a given speed *v*. Furthermore, common driver behaviour observations show that drivers never accelerate beyond $50\%$ of the maximum acceleration capacity of the vehicle for the given speed^54^. These vehicle and driver dynamics constraints are illustrated via the speed-acceleration curve of an exemplary vehicle in Fig. 3b. Hard thresholding is commonly performed to enforce the vehicle dynamics constraints; however, it alters distances travelled by the vehicles when faced with infeasibly large accelerations and, hence, reduces the physical consistency at both individual vehicles and platoon levels. Therefore, the optimization problem (6) combines these aforementioned objectives together with restoring the physical consistency to the reconstructed trajectories.

The cost function in (6) balances noise reduction and driver compliance objectives within the trajectory reconstruction problem. Specifically, the first term in (6a) reduces the noise in the reconstructed vehicle speed where $V_{\text {filt}}$ is obtained by applying a first-order Butterworth filter with a cut-off frequency determined by the method developed in^45^. The noise reduction cost weight is denoted by $\lambda _N$. The second term in (6a) minimizes the acceleration variance within the reconstruction window; whereas the third term penalizes violations beyond the acceleration capacity of the common driver in a soft constraint form. We set the driving style $d = 0.5$, and $\lambda _A$ and $\lambda _{DC}$ denote the cost weights for acceleration variance and driver compliance objectives. Therefore, these terms ensure the reconstructed trajectories comply with common driver dynamics.

The first constraint (6b) requires the reconstructed vehicle speed to be non-negative. The constraint (6c) defines the vehicle dynamics constraints as follows

7 $$\begin{aligned} d_{\text {min}} = \max (d_p(v(k)), d_p(\mu _{V_{\text {filt}}})), \qquad a_{\text {max}} = \max (a_p(v(k)), a_p(\mu _{V_{\text {filt}}})) \end{aligned}$$

where $\mu _{V_{\text {filt}}}$ denotes the mean filtered speed within the reconstruction window and *v*(*k*) denotes the original initial speed in the reconstruction window. Jerk constraints may also be included here. The third and fourth constraints (6d)–(6e) ensure physical consistency of the reconstructed trajectories both in the internal and platoon sense. Specifically, the constraint (6d) ensures that the reconstructed speed trajectory of the ego vehicle covers the same travelled distance $x(k+w_{\text {recon}}+1) - x(k)$ within the reconstruction window. The last constraint (6e) ensures a minimum space gap of $s_{\text {min}} = 1$ m between the preceding and the ego vehicles; where $x_{\text {prec}}$ and $L_{\text {prec}}$ represent the position and length of the preceding vehicle. This prevents unrealistic space headways (e.g. negative space headways, overtaking, etc.). This is especially true in our conducted car-following experiments.

Once the optimization problem is solved, the reconstructed speed $V_{\text {recon}}$ is used to compute the reconstructed position $X_{\text {recon}}$ and acceleration $A_{\text {recon}}$ via Euler integration and forward difference respectively. Then, the sliding window is moved via $w_{\text {step}}$ into the next overlapping reconstruction window, and the procedure is repeated till the entire ego vehicle trajectory is reconstructed and we move on to the following vehicle. For our setup, determining the first vehicle to start the reconstruction procedure is not necessarily stringent due to the circular track nature. However, this circular dependency does not entail an iterative approach since the knowledge of the circular track diameter allows us to disentangle this circular dependency.

The relaxed optimization problem for trajectory reconstruction is expressed as follows.

8a $$\begin{aligned} V_{\text {recon}}^\star = \arg&\min _{V_{\text {recon}}} \frac{\lambda _N}{w_{\text {recon}}+1}\left\Vert V_{\text {recon}} - V_{\text {filt}} \right\Vert _2^2 + \frac{\lambda _A}{w_{\text {recon}}} \left\Vert A_{\text {recon}} - \mu _{A_{\text {recon}}} \right\Vert _2^2 \nonumber \\&+ \lambda _{DC} \sum _{\tau =k}^{k+w_{\text {recon}}-1} \Bigg ( \frac{a_{\text {recon}}(\tau )}{d a_{\text {max}}}-1 \Bigg ) + \lambda _1 \epsilon _{\text {min}}^2 + \lambda _2 \epsilon _{\text {max}}^2 \end{aligned}$$

8b $$\begin{aligned} \text {s.t. }&V_{\text {recon}} \ge 0 \end{aligned}$$

8c $$\begin{aligned}&A_{\text {recon}} - d_{\text {min}} \ge \epsilon _{\text {min}}, \quad \epsilon _{\text {min}} \le 0 \end{aligned}$$

8d $$\begin{aligned}&A_{\text {recon}} - a_{\text {max}} \le \epsilon _{\text {max}}, \quad \epsilon _{\text {max}} \ge 0 \end{aligned}$$

8e $$\begin{aligned}&x(k+w_{\text {recon}}+1) - x(k) = \Delta t \sum _{\tau =k}^{k+w_{\text {recon}}} v_{\text {recon}}(\tau ) \end{aligned}$$

8f $$\begin{aligned}&x_{\text {prec}}(\eta +1) - L_{\text {prec}} - \Big ( x(k) + \Delta t \sum _{\tau =k}^{\eta } v_{\text {recon}}(\tau ) \Big ) \ge s_{\text {min}}, \quad \forall \eta \in \{ k, \dots k+w_{\text {recon}} \} \end{aligned}$$

where the vehicle dynamics constraints are relaxed in (8c)–(8d) using the relaxation variables $\epsilon _{\text {min}}$ and $\epsilon _{\text {max}}$. This relaxed optimization problem (8) is used for benchmarking the different OBB and HBB models in the aforementioned results; where the magnitude of the relaxation variables indicate the degree of dissatisfaction of the vehicle dynamics constraints in the reconstruction process and therefore the reconstructability of the extracted trajectories.

### Aerial video recording data

Aerial videos from a real world experiment were used for the benchmark and evaluation. These videos were recorded during an experiment with 14 (15) vehicles of different size, colour, and shape, that were driving on a circular test road as shown in Fig. 1. The experiment was conducted on 12th of March 2024, with drone model *DJI AIR 2S*, as part of a production for the Swiss television *SRF* at the *TCS Driver Training Center Derendingen* in Switzerland. The video material has a resolution of 3840 x 2160 pixels, a rate of 25 frames per second, and includes 40 minutes of recording (50 GB). The aerial perspective and the circular lane are ideal for demonstrating performance gains when extracting vehicle trajectories using oriented object detection. Moreover, a circular, closed-loop track allows for the observation of numerous vehicle interactions with just one drone, on an “infinitely-long” highway without any road-geometric interference. A list of vehicles and their specifications that took part in the experiment can be found in Table 2.

**Table 2:** Vehicle information table.

Nr.	Color	Pos.	Brand	Model	Year	Powertrain	Gearbox	ADAS	Displ. [l]	Power [HP]	Torque [Nm]	Mass [kg]
1	black	[1]	SEAT	Alhambra II	2022	Combustion	Automatic	yes	2.0	150	340	1,800
2	gray	[2]	Toyota	BZ 4 X	2022	Electric	Automatic	yes	-	218	266	1,920
3	darkgray	[3]	Renault	Laguna III	2015	Combustion	Manual	no	2.0	150	340	1,630
4	silver	[4]	VW	Scharan TSI	2017	Combustion	Automatic	no	1.4	150	250	1,790
5	silver	[5]	Renault	Laguna III	2014	Combustion	Automatic	no	2.0	150	340	1,630
6	gray	[6]	Toyota	Verso D-4D	2015	Combustion	Automatic	no	2.2	177	400	1,530
7	red	[7]	Citroen	Picasso C3 VTi 120 EGS6	2015	Combustion	Manual	no	1.6	120	160	1,310
8	black	[8]	Ford	Focus NA3	2018	Combustion	Manual	no	1.5	150	240	1,360
9	beige	[9]	VW	Caddy Maxi TSI	2019	Combustion	Automatic	yes	1.4	125	200	1,450
10	lightblue	[10]	Volvo	Polestar 1, Raceversion	2021	Combustion	Automatic	yes	2.0	600	1,000	2,350
11	white	[11]	VW	Caddy 2K TSI	2012	Combustion	Manual	no	1.2	86	160	1,450
12	black	[12]	Peugeot	RCZ R	2015	Combustion	Manual	no	1.6	270	330	1,340
13	gray	[13]	VW	Golf, Gold VII Typ AU	2014	Electric	Automatic	yes	-	115	270	1,240
14	white	[14]	Audi	A4 TDI DSG	2016	Combustion	Automatic	yes	2.0	190	400	1,480
**Additionally inserted in experiment 3:**												
15	black	[after 2]	Opel	Adam 1.4	2019	Combustion	Manual	no	1.4	87	130	1,060

## Results

The vehicle trajectory extraction from aerial recordings follows certain steps of a proposed, computational pipeline, as outlined in Fig. 1. A selection of OBB and HBB models (Table 1) are employed when the extraction method is applied to a real-world aerial recording. The performance of different OBB and HBB models are analysed for each step of the pipeline in the following, to identify significant differences between oriented (OBB) object detection and horizontal (HBB) object detection, and to benchmark these models.

### Object detection and annotation filtering

**Figure 4:**
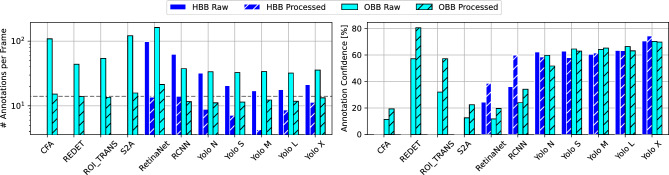
Object detection annotation quality.

Figure 4 summarizes the quality analysis of the object detection (of vehicles on single frames). The number of annotations per frame (left plot) represents how many objects were detected on a single frame of the video. The number of objects on the videos was between 14 and 15 vehicles (dashed line). The dark-blue bars represent the number of vehicles identified by the HBB models, and the light-blue bars represent the number of vehicles identified by the OBB models. Non-hatched bars depict the number of annotations before annotation filtering, and hatched bars after filtering. All models find more objects in the frames than there are cars before filtering (on average 38 objects for HBB and 63 objects for OBB). After the filtering (exclusion of infeasible annotations), most models find a number of objects close to the number of actual vehicles (on average 9 for HBB and 13 objects for OBB). Due to the larger number of initial guesses for OBB models, that are inherently trained to detect oriented objects from aerial perspectives, the remaining number of objects found after filtering matches more closely the number of actual vehicles. Therefore, OBB models are able to provide qualitatively better annotations for the next steps of the pipeline, as on average almost every car is annotated on each frame of the video. The average confidence of annotations per frame (right plot) leads to a similar conclusion. The neural network models provide a confidence for each annotation, reflecting how sure the model is about each of its findings. On average, the OBB models have a confidence of 43 % per annotation before filtering, and 49% after filtering. The HBB models have a confidence of 54% per annotation before filtering, and 59% after filtering. This indicates, that OBB models are more critical towards their findings and performing better, while HBB models are overconfident, leading to the provision of qualitatively worse annotation for the next steps of the trajectory extraction process.

### Trajectory building and matching quality

**Figure 5:**
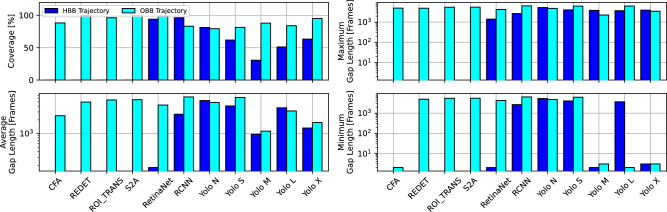
Trajectory building, matching quality and gap analysis.

Figure 5 describes the matching quality during the trajectory building step (matching annotations to vehicles across frames, resulting in an annotation time series for each vehicle). The coverage of trajectories (share of frames where each vehicle is annotated) demonstrates, that due to the better annotations from OBB models (both in quantity and quality) leads to a higher coverage of vehicles of around 90%, while the HBB models achieve only 68% coverage on average. Besides of solely analysing the coverage of trajectories, the analysis of the gap lengths in the vehicles’ time series is shown in the other plots of the figure. While OBB models have a higher coverage, the few gaps are of a length of 2,058 frames (on average), 1,733 frames (at minimum), and 2,886 frames (at maximum). The HBB models on the other hand, have shorter gaps of a length of 1,910 frames (on average), 1,591 frames (at minimum), and 2,229 frames (at maximum). The shorter gaps of HBB models can be beneficial when using the Kalman Filtering in the next step to fill the time series’ gaps. However, the difference in gap lengths of OBB and HBB models is significantly smaller when compared with the previous quality assessments. Moreover, amongst the best performing models (shortest gaps on average, at minimum, and at maximum) we can observe OBB models rather than HBB models.

**Figure 6:**
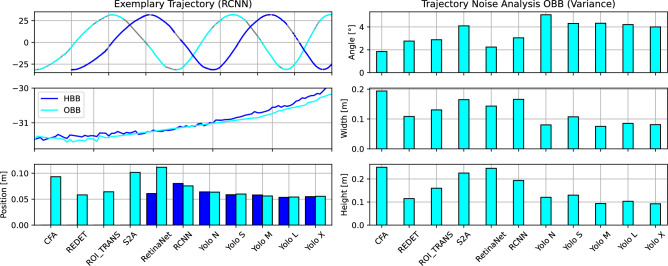
Trajectory state noise.

To further assess the trajectory quality of the trajectory building step, we analysed the noise levels of different trajectory states, as shown in Fig. 6. The two upper plots of the left column display the Cartesian X coordinate of single vehicles driving in the circle over time. The upper plot shows the covered frames (in dark-blue and light-blue, representing HBB and OBB), and where gaps (in grey) are located in the vehicle trajectory’s time series. The lower plot shows the trajectory of the same vehicle but zoomed in, and reveals that the time series possesses some noise. Therefore, the noise levels (measured in variance between consecutive frames) for different trajectory states (position, angle, width, height) are analysed in the other plots. HBB models only provide (Cartesian) positional state information. The OBB models can additionally provide angle, width, and height due to the oriented bounding boxes. Larger noise levels can negatively affect the quality of the final trajectories. The comparison of HBB and OBB models (for all models where the comparison was possible) shows a negligible difference in positional noise (0.611 m for HBB, 0.068 m for OBB). An exception is the model RetinaNet, where significantly higher noise levels for the OBB version can be observed. With regards to the other states, large differences in the state noise levels across different OBB models can be observed. Yet, the noise levels for the different vehicle states are relatively small overall (0.16% for position, 1.11% for angle, 6.67% for width, 4% for height).

### Kalman filter and trajectory quality

**Figure 7:**
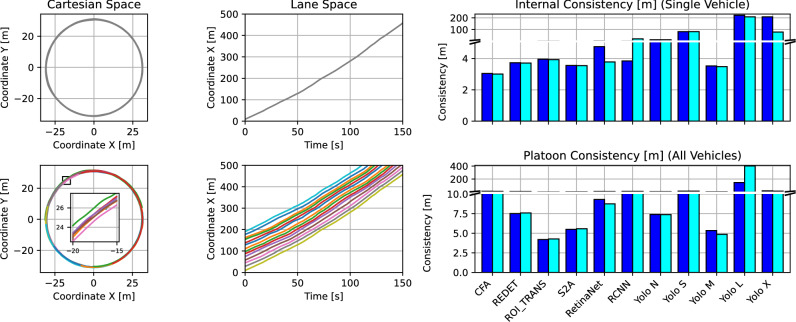
Trajectory quality: internal and platoon consistency.

Figure 7 assesses the quality of the trajectories after Kalman Filtering, and translation into lane coordinates. The Kalman Filter provides a filtered time series, where gaps are filled (imputation), and additional vehicle states (velocity, acceleration, angular velocity). The internal and platoon consistency^24^ measures assess the quality of the Kalman-filtered time series, by comparing integrated acceleration and velocity with the vehicle’s progressed distance in lane coordinates. As a result, physically more plausible trajectory states should result in lower deviations from lane coordinates (lower consistency measure means better). Internal consistency is calculated for a single vehicle, while platoon consistency is calculated by aggregating a platoon of all vehicles together (considering inter-vehicle space and time headways). To provide context for comparing such error measures with existing datasets, the authors in^24^ define a reliable dataset as one with mean absolute consistency errors below 1 m. Furthermore, the internal and platoon consistency errors for the NGSIM dataset, for instance, had worst-case values up to 691.2 m and 59.3 m, and mean values up to 3.35 m and 0.04 m respectively. It should also be noted that the NGSIM dataset displayed up to 80% and 23.5% of total vehicles with internal and platoon consistency errors outside the defined reliability range^24^.

Certain models had very large gaps that could not be Kalman-filtered well enough, leading to very large deviations when compared to the other models (RCNN, Yolo S, Yolo L, Yolo X). On average, the internal consistency error of OBB models was 38.75 m, and of HBB models was 50.31 m. When excluding the outlying models, the internal consistency error of OBB models was 4.99 m, and of HBB models was 5.15 m. On average, the platoon consistency error of OBB models was 44.72 m, and of HBB models was 22.24 m. When excluding the outlying models, the platoon consistency error of OBB models was 7.16 m, and of HBB models was 7.44 m. As a result, OBB models achieve up to 22.98% improvement in internal consistency (3.11% excluding outlying models), and up to 3.76% improvement in platoon consistency (excluding outlying models). Therefore, we conclude that an angle-informed Kalman Filter (OBB) is enabled to produce better (more consistent) trajectory when compared with HBB.

Table 4 systematically benchmarks the internal and platoon consistency errors (maximum, minimum, average, and standard deviation) for all models investigated. Models 1 to 11 represent OBB models; these models can pass the Kalman Filter using the available angle information or not. Models 12 to 18 represent HBB models; these models can only pass the Kalman Filter without angle information. When integrating acceleration and velocity states, one can determine the inconsistency errors with lane coordinates.

The standard deviation of internal consistency errors of OBB models was 170.66 m, and of HBB models was 239.33 m. When excluding outlying models, the internal consistency error of both OBB and HBB models was 23.55 m. The standard deviation of platoon consistency errors of OBB models was 57.48 m, and of HBB models was 65.11 m. When excluding outlying models, the platoon consistency error of OBB models was 8.87 m, and of HBB models was 8.98 m. As a result, OBB models achieve up to 28.69% less variation in internal consistency errors, and up to 11.72% less variation in platoon consistency errors (1.22% excluding outlying models). This is another indication for the conclusion, that an angle-informed Kalman Filter (OBB) is enabled to produce better (more consistent) trajectory when compared with HBB.

**Figure 8:**
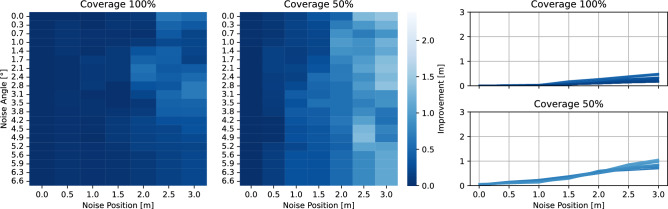
Trajectory quality: internal consistency of synthetic trajectories.

To analyse the role of state noise when improving trajectory consistency using OBB, we furthermore conducted experiments with synthetic trajectories. We generated synthetic trajectories of cars driving in circles, with different, Gaussian-distributed noise levels in position and angle, and different coverages. Figure 8 exhibits the results of this analysis. The two heatmaps depict the improvement of internal consistency using orientation-informed Kalman Filtering (OBB) over HBB models, where light blue stands for greater improvements. Especially, when the coverage is low (50%, trajectories have gaps), and the positional information is noisy, the usage of OBB can create significant improvements. These improvements however depend on the quality of the angle information; for increasing angle noise, the improvements diminish, as the value of the angular information decays. The improvements in internal consistency can go as high as 3 m (2%). To summarize, the angular information provided by OBB generates improvements, especially when it is challenging for HBB filtering due to noise in position or issues with coverage.

### Trajectory reconstruction plausibility

**Table 3:** Validation of utilized trajectory reconstruction algorithm via internal and platoon consistency measures.

	Internal Consistency [m]				Platoon Consistency [m]				Total Distance Travelled [m]	Physically Implausible Space Headways [%]
	MIN	MAX	AVG	STD	MIN	MAX	AVG	STD		
Before reconstruction	0	8.86	3.54	2.41	0	19.0	5.58	4.33	1133.12	0
Proposed reconstruction	0	0.20	0.06	0.04	0	0.34	0.09	0.08	1133.25	0
Butterworth reconstruction	0	0.09	0.03	0.02	0	0.17	0.06	0.04	1128.80	6.09
Wavelet-based reconstruction	0	0.19	0.07	0.04	0	0.30	0.08	0.07	1133.11	0

**Figure 9:**
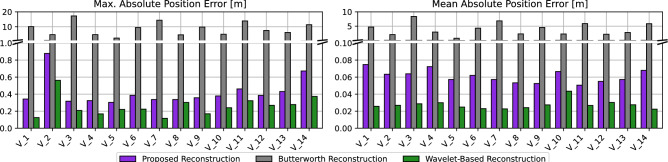
Comparison of changes in lane-coordinate positions per vehicle before and after reconstruction.

To enhance the consistency of vehicle trajectories, one can apply convex optimization approaches to post-process the states altogether in order to derive reconstructed variables, that minimize consistency measures. First, we demonstrate and validate the utilized trajectory reconstruction algorithm against a state-of-the-art method by^55^ and a baseline method for the OBB model *S2A*. The state-of-the-art method uses wavelet analysis to filter the vehicles’ positional trajectories using a simple two-step approach and it will be termed as “wavelet-based reconstruction” thereafter. As for the baseline method, it uses a Butterworth filter with a cut-off frequency of 0.75 Hz; hence, it will subsequently be referred to as “Butterworth reconstruction”.

Table 3 illustrates the internal and platoon consistency measures before reconstruction and after reconstruction using the proposed, Butterworth and wavelet-based reconstruction approaches. We can observe that all trajectory reconstruction approaches successfully filter the vehicles’ trajectories and minimize the consistency error measures. However, the Butterworth reconstruction method alters the distance travelled by the vehicles; thus, resulting in implausible space headways (i.e. crashes that did not occur in the original experiment). The underlying reason is that the filtered speed does not consider the ensuing changes in the rest of the trajectory variables. Regarding the wavelet-based reconstruction method, it minimally alters the vehicles’ positional trajectories and minimizes the consistency error measures. To further demonstrate said changes in the vehicles’ positional trajectories, Fig. 9 compares the maximum and mean absolute changes in lane-coordinate positions for both reconstruction methods. On average, the proposed and the wavelet-based reconstruction algorithms only alter the lane-coordinate position of the vehicles within 0.06 m and 0.03 m respectively; whereas the Butterworth reconstruction alters within 4 m. Therefore, the proposed reconstruction approach circumvents these issues through the constrained optimization problem used. It is also worth noting that both the proposed and wavelet-based reconstruction methods achieve very similar consistency error measures, even though the proposed algorithm allows for slightly more changes in the vehicles’ positional trajectories, as observed in Fig. 9. These slightly higher positional changes allow the proposed reconstruction method to enforce the vehicle and driver dynamics constraints, which we will demonstrate next.

**Figure 10:**
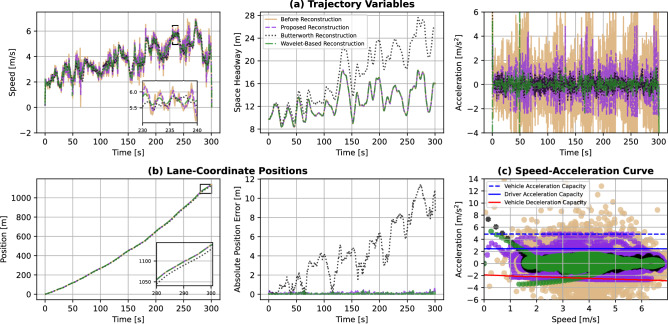
Comparison of the reconstructed trajectory of Vehicle 1.

**Figure 11:**
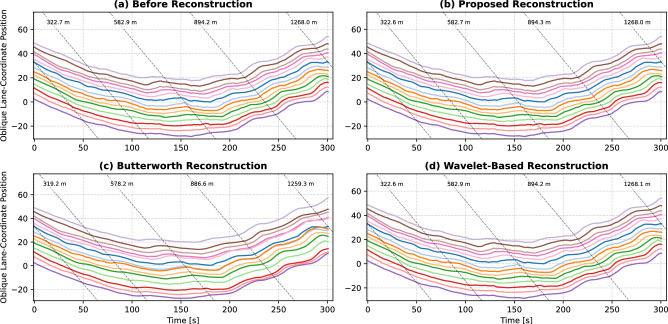
Comparison of the reconstructed oblique trajectories of all vehicles.

An exemplary vehicle trajectory is shown before and after reconstruction in Fig. 10. We can observe the ensuing changes in the space headway and lane-coordinate position for the Butterworth reconstruction method. As for the wavelet-based method, it does not explicitly enforce the vehicle dynamics constraints, but the acceleration is filtered indirectly through the choice of the mother wavelet in the noise reduction step of the algorithm. Therefore, unrealistic accelerations are recorded after wavelet-based reconstruction as shown in Fig. 10c. On the other hand, the proposed approach keeps these position changes to a minimum, while also satisfying both the vehicle and driver dynamics constraints as illustrated in Fig. 10c. Hence, the proposed reconstruction algorithm is able to provide physically consistent trajectories in a vehicle- and driver-informed manner. Figure 11 shows the vehicles’ oblique trajectories for all vehicles on the test track during a 300-second experiment run. It can be observed again that all the vehicles’ trajectories did not substantially change after the reconstruction using the proposed algorithm. On the other hand, the Butterworth reconstruction method yields not only changes in the vehicles’ trajectories, but also some crashes between adjacent vehicles that did not occur in reality.

**Figure 12:**
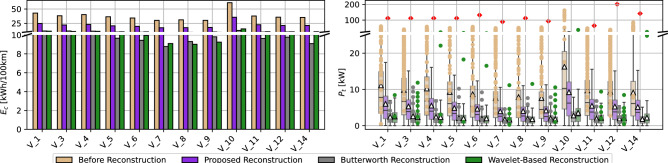
Comparison of the tractive energy consumption of all fuel-engine vehicles before and after trajectory reconstruction. In the right figure, the red diamonds represent the vehicles’ maximum power (see Table 2), and white triangles indicate the recorded mean power. Outlier power values (i.e. above the speed-dependent maximum tractive power) are indicated by the filled circles per reconstruction method.

The incorporation of vehicle and driver dynamics is also beneficial for energy consumption studies using the reconstructed trajectories (Fig. 12). The tractive power $P_t$ and energy $E_c$ consumption are computed according to ^?^. Also, the maximum tractive power can be computed similarly using the vehicle’s acceleration capacity from the MFC model at each given speed. We can observe unrealistic or outlying power values that are above the vehicles’ capabilities (i.e. above the maximum tractive power according to vehicles’ specifications at the operating speed) before reconstruction. All three reconstruction methods yield tractive power below the vehicles’ maximum power capacity specified in Table 2. However, both the Butterworth and wavelet-based reconstruction methods highly suppress the tractive power and hence energy consumed by each vehicle. This might be unrealistic and can be detrimental to the trajectory quality and usability. It should also be noted that they do not eliminate outlier tractive power values that are above the speed-dependent maximum tractive power. This is owing to the lack of enforcing the vehicle and driver dynamics constraints within these reconstruction methods. On the contrary, the proposed method tends to maintain a wider distribution of tractive power and consequently higher energy consumed after reconstruction. We deem these results by the proposed reconstruction method realistic due to the satisfaction of the vehicles’ dynamics, the maximum power specifications and the speed-dependent maximum tractive power. Therefore, the physics information imbued in the algorithm promotes the consistency and plausibility of the reconstructed trajectories and enhances their usability in various subsequent studies.

**Figure 13:**
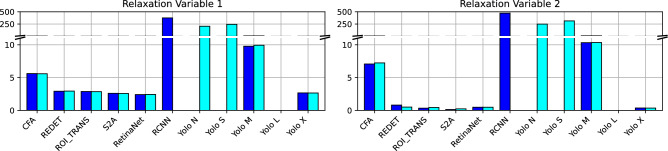
Reconstructability of trajectories.

Now, in order to compare the different object detection models, we employ a relaxed version of the constrained optimization problem in the proposed algorithm. These two relaxation variables transform the acceleration constraints capturing the vehicles’ capabilities into soft constraints. The stronger these relaxation variables are used during reconstruction, the stronger the Kalman-filtered trajectories were manipulated and violated minimum (RLX_1 or $\left\Vert \epsilon _{\text {min}} \right\Vert _{\infty }$) and maximum (RLX_2 or $\left\Vert \epsilon _{\text {max}} \right\Vert _{\infty }$) acceleration constraints. Figure 13 and Table 4 display the magnitude of the relaxation variables. The reconstruction failed for several models (RCNN, Yolo N, Yolo S, YOLO L) for numerical reasons. First, one can observe that the number of models, for which a trajectory reconstruction was possible, is greater for OBB models instead of HBB models. Second, the magnitude of relaxation variables is almost similar for HBB models (4.14 for RLX_1, 2.80 for RLX_2) and OBB models (4.12 for RLX_1, 2.79 for RLX_2), excluding the outliers. In addition, we can observe that more OBB models produce reconstructable trajectories than HBB models. Hence, we can conclude that OBB models produce more plausible trajectories than HBB models; which can be further enhanced through the proposed reconstruction procedure in terms of physical consistency on both the individual vehicle and platoon levels.

### Model benchmark

With regards to benchmarking all models included, and determining a recommendation for model choice, let us review Table 4 again. The top performing model in internal consistency (*CFA*) is not the best performing model in platoon consistency (ROI_TRANS). The second best models however (*S2A* and *Yolo M*) perform nearly well but consistently across internal and platoon consistency.

Considering the performance across all steps of the pipeline, and the final results in Table 4, we recommend *S2A*, as this model performs well across both consistency measures, and reconstruction feasibility. Besides, the models *CFA*, *REDET*, *ROI_TRANS*, *RetinaNet*, and *RetinaNet* delivered good results as well. The findings for the *Yolo* model family are mixed, where *Yolo M* performed best.

**Table 4:** Optical trajectory extraction benchmark.

			HBB (angle unavailable)						OBB (angle available)					
N	Model	Type	MAX	MIN	AVG	STD	RLX_1	RLX_2	MAX	MIN	AVG	STD	RLX_1	RLX_2
			**Internal consistency**											
1	CFA	OBB	8.71	0.00	3.04	2.02	− 5.61	7.07	8.70	0.00	3.00	1.99	− 5.59	7.24
2	REDET	OBB	9.12	0.00	3.72	2.52	− 2.92	0.81	9.07	0.00	3.71	2.51	− 2.94	0.51
3	ROI_TRANS	OBB	9.83	0.00	3.95	2.68	− 2.88	0.34	9.77	0.00	3.92	2.67	− 2.86	0.44
4	S2A	OBB	8.89	0.00	3.55	2.42	− 2.60	0.14	8.86	0.00	3.54	2.41	− 2.58	0.24
5	RetinaNet	OBB	9.66	0.00	3.78	2.66	− 2.41	0.48	9.62	0.00	3.77	2.65	− 2.40	0.48
6	RCNN	OBB	57,405.44	0.00	21.10	688.18	− 375.36	471.13	57369.39	0.00	21.19	687.75		
7	Yolo N	OBB	12,374.07	0.00	13.51	150.05			12383.91	0.00	13.51	150.17	− 204.92	249.11
8	Yolo S	OBB	28,966.23	0.00	82.64	416.65			29011.86	0.00	82.33	398.95	− 244.56	313.73
9	Yolo M	OBB	9.68	0.00	3.52	2.47	− 9.78	10.32	9.49	0.00	3.48	2.43	− 9.93	10.35
10	Yolo L	OBB	103,168.10	0.00	222.25	1356.57			44729.96	0.00	208.61	619.40		
11	Yolo X	OBB	85.56	0.00	79.33	6.37	− 2.67	0.36	85.44	0.00	79.23	6.36	− 2.65	0.35
12	RCNN	HBB	9.51	0.00	3.84	2.59	− 2.39	0.40						
13	RetinaNet	HBB	11.33	0.00	4.76	3.25	− 2.42	0.45						
14	Yolo N	HBB	5951.09	0.00	9.64	105.31								
15	Yolo S	HBB	43,906.07	0.00	60.04	613.12								
16	Yolo M	HBB	273,026.71	0.00	300.66	3742.69								
17	Yolo L	HBB	28,469.45	0.00	71.85	495.45								
18	Yolo X	HBB	39,27475.49	0.00	570.28	45747.25								
			**Platoon consistency**											
1	CFA	OBB	38.57	0.00	12.82	8.74	− 5.61	7.07	36.91	0.00	11.75	8.35	− 5.59	7.24
2	REDET	OBB	22.88	0.00	7.51	5.19	− 2.92	0.81	23.30	0.00	7.58	5.28	− 2.94	0.51
3	ROI_TRANS	OBB	15.32	0.00	4.20	3.49	− 2.88	0.34	15.63	0.00	4.27	3.55	− 2.86	0.44
4	S2A	OBB	26.96	0.00	9.31	6.14	− 2.60	0.14	25.84	0.00	8.75	5.88	− 2.58	0.24
5	RetinaNet	OBB	18.71	0.00	5.50	4.26	− 2.41	0.48	19.00	0.00	5.58	4.33	− 2.40	0.48
6	RCNN	OBB	12,577.72	0.00	11.59	156.58	− 375.36	471.13	12573.21	0.00	11.59	156.67		
7	Yolo N	OBB	2194.31	0.00	7.39	30.83			2196.07	0.00	7.37	30.88	− 204.92	249.11
8	Yolo S	OBB	7853.50	0.00	14.23	105.05			7123.42	0.00	13.75	96.54	− 244.56	313.73
9	Yolo M	OBB	18.24	0.00	5.34	4.22	− 9.78	10.32	16.36	0.00	4.85	3.78	− 9.93	10.35
10	Yolo L	OBB	16,685.95	0.00	147.28	295.32			10,908.30	0.00	402.00	391.55		
11	Yolo X	OBB	55.34	0.00	19.47	12.45	− 2.67	0.36	41.14	0.00	14.42	9.40	− 2.65	0.35
12	RCNN	HBB	299.13	0.00	80.19	68.31	− 2.39	0.40						
13	RetinaNet	HBB	21.87	0.00	6.88	4.93	− 2.42	0.45						
14	Yolo N	HBB	3876.99	0.00	14.34	60.27								
15	Yolo S	HBB	11,285.88	0.00	187.88	214.11								
16	Yolo M	HBB	13,4864.18	0.00	1271.05	2220.71								
17	Yolo L	HBB	20,507.93	0.00	45.39	321.47								
18	Yolo X	HBB	56,8775.05	0.00	140.39	6670.78								

### Case study: string stability analysis

**Figure 14:**
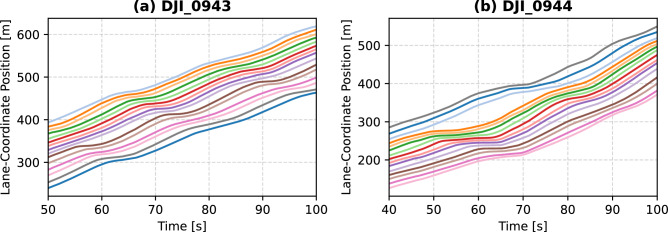
Snippet of reconstructed vehicles’ trajectories for two videos where considerable disturbances are present.

To demonstrate the usefulness of the proposed trajectory filtering and reconstruction methods, the presented car-following experiment is leveraged to analyse string stability of the vehicle platoon. First, we extracted interesting snippets, shown in Fig. 14, from the dataset where significant disturbances occur and propagate upstream.

**Figure 15:**
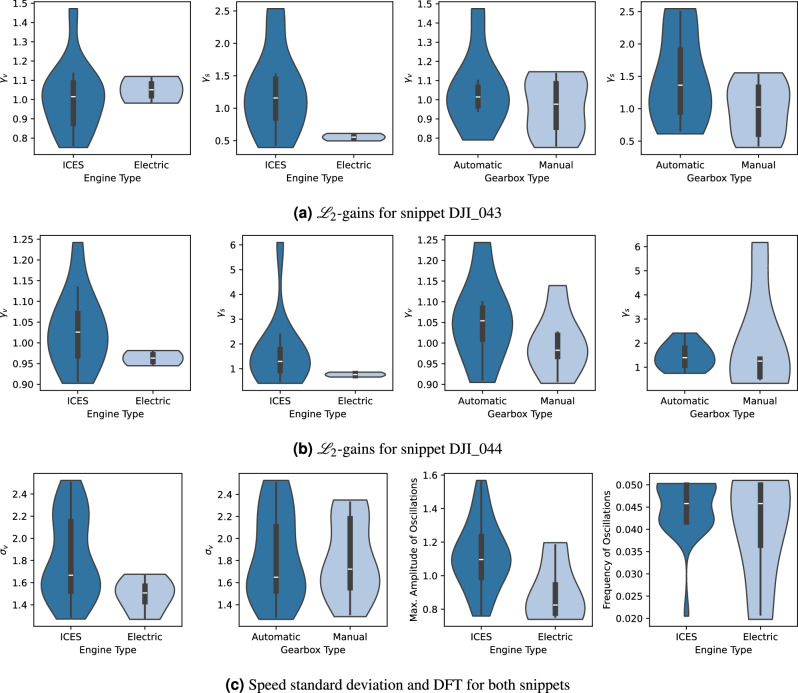
Analysis metrics for the reconstructed vehicles’ trajectories during snippets with considerable disturbances. “ICES” stands for internal combustion engine system.

Figure 15 illustrates the different analysis metrics used for the aforementioned vehicles’ trajectory snippets. The first metric is the estimated $\mathscr {L}_2$-gain of each leader-follower subsystem. This metric measures the amplification/reduction gain of perturbations from the leader to the follower vehicle’s states. This gain can be computed for the speed and space headway states, denoted by $\gamma _v$ and $\gamma _s$ respectively. This computation is based on a semi-definite program as explained in^56^. The second metric is the standard deviation of the vehicles’ speed, $\sigma _v$. The last metrics, the maximum amplitude and frequency of oscillations, are obtained from discrete Fourier transform (DFT) after applying a Hanning window on the de-trended speed profile^57^.

It can be observed from Fig. 15a,b that $\gamma _s$ is consistently lower for electric engines than Internal Combustion Engine Systems (ICES). This can be attributed to smoother speeds for electric engine systems as evidenced by lower $\sigma _v$ and lower oscillation amplitude in Fig. 15c. The speed standard deviation does not differ much per gearbox type. The underlying reason may be that the driving style and gear-shifting style of the driver are the deciding factors for that. Regarding the speed-related $\mathscr {L}_2$-gains, Fig. 15b shows a lower $\gamma _v$ for electric engine systems than ICES. This is evident for snippet *DJI_044* since the disruption is of a higher magnitude, as observed in Fig. 14b. On the other hand, for snippet *DJI_043*, $\gamma _v$ for both electric and ICES are similar on average where the perturbations are not as strong as in the latter case.

## Discussion

Trajectory extraction pipelines usually use HBB-based object detection models. HBBs often include complex backgrounds and can struggle with detecting arbitrarily oriented objects, while OBBs more accurately enclose objects, reducing background noise and occlusion. Consequently, OBBs are more suitable for applications requiring precise object orientation, such as vehicle trajectory estimation and driver behaviour analysis. Hence, the contribution of this work is twofold. First, we systematically analyse the use of OBB and compare its performance gains with HBB models. Second, we propose a robust vehicle trajectory extraction pipeline, that leverages angular information if available.

**OBB versus HBB models.** The results highlight that OBB models consistently and critically capture the vehicles’ annotations per frame compared to HBB models. Therefore, using OBB models leads to better performance in the subsequent steps of the designed pipeline; for instance, higher coverage and lower gap lengths in the vehicles’ time series. In addition, the lower gap lengths give rise to higher fidelity of the Kalman-filtered trajectories. This was illustrated using the physical consistency measures of the trajectories; both on the individual vehicle level and the platoon level. The OBB models achieved around 22.98% and 3.76% improvement in internal and platoon consistency compared to HBB models; thereby showcasing the added benefit of angle information to the extraction pipeline. Furthermore, the synthetic noise analysis has illustrated that OBB models perform significantly better even in the presence of low coverage and noisy positional data. However, these improvements naturally decrease with the increase of the noise in the angular data.

**Trajectory reconstruction.** Trajectory reconstruction is performed in order to obtain not only physically consistent trajectories, but also vehicle- and driver-compatible ones. Notably, hard thresholding of infeasible accelerations is avoided through the use of a sliding-window constrained optimization approach. This sidesteps the need for large changes made to the vehicles’ positions which may result in unrealistic behaviours (e.g. crashes). Hence, the vehicles’ trajectories are minimally adjusted to satisfy vehicle and driver dynamics constraints. Also, such dynamics constraints are naturally non-linear and vary for different engine types (i.e. internal combustion, electric, hybrid) and this is captured within the reconstruction algorithm; allowing for retaining the real observed driving behaviour and characteristics. The assumption imposed here is the knowledge of the vehicles’ specifications to determine said vehicle dynamics constraints. In large-scale experiments, this information may not be available; however, averaged models^58^ may be beneficial for such use cases. Furthermore, trajectory reconstruction could be improved using visual spatio-temporal maps^59,60^ in order to isolate the positional errors from the vehicle’s actual distance travelled. In our reconstruction algorithm, this is achieved via the imposed constraints. Future work could explore the integration of visual spatio-temporal maps to correct/reconstruct vehicles’ trajectories earlier in the extraction pipeline. A relaxed trajectory reconstruction approach is then utilized to compare the usage of OBB and HBB models. OBB models require less relaxation of the imposed constraints in the trajectory reconstruction algorithm. This can be attributed to the higher plausibility of the OBB-based trajectories which, in turn, are more feasible for and can be enhanced through reconstruction.

**String stability case study.** A case study was presented on two snippets of vehicles’ trajectories from the conducted experiment to showcase the usefulness of the recorded and reconstructed dataset. Here, the case study was to analyse the string stability for the conducted car-following experiment. Further use cases may include calibration of car-following models and analysis of traffic flow patterns. The uniqueness of this dataset lies within its mixed platoon nature (i.e. different power-train and gearbox types). The analysis metrics have shown that electric engine systems are capable of smoother speed profiles; observed by the lower speed standard deviations and maximum amplitude of oscillations. This may allow for more string-stable leader-follower subsystems; as measured by the estimated $\mathscr {L}_2$-gains. It is important to note that these observations are made in the low-speed range (i.e. 0–10 m/s); where the driver behaviour may influence the perturbation evolution more prominently than engine or gearbox types. This highlights the need for more experimental campaigns with mixed-vehicle platoons to understand the impact of vehicle dynamics on stop-and-go wave formation and propagation for medium to high speed ranges. For our setup, the small diameter of the circular track (around 65 m) did not allow for higher speed ranges during the conducted experiment. Still, our analysis suggests that electric engine systems give rise to smoother traffic flows. Driver behaviour is an important factor for stable traffic flows; yet, electric engine systems may allow for a smoother driving experience and improve reaction times. This is an interesting point for future research and experimental campaigns to discuss these interactions between engine and driver types.

**Pipeline generalizability.** This pipeline was demonstrated at the example of a circular, single-laned track. This significantly simplified (i) homography (translation of image coordinates to Cartesian coordinates), (ii) coordinate transforms (translation of Cartesian coordinates to lane coordinates), (iii) observation of all vehicle interactions with just one drone / camera, and (iv) tracking vehicles. This work could be easily be applied to other experimental setups. First, for homography, notable image markers as reference points could be used in order to locate the vehicles with respect to known coordinates within the image for instance. In the context of this study the circular pattern was simple to extract from the videos, but in more complex contexts and larger spatially distributed applications, multiple, printed QR codes or other visual marking points could be used for homography. Second, for coordinate transforms, a conversion from Cartesian coordinates, over polar coordinates, to Lane coordinates was simple due to the circular shape of the road geometry. Future works on more complex geometries might separate the road into certain splines, to conduct such transforms (see Fig. 2). Third, for larger areas to be observed, multiple drone / camera recordings become necessary. This requires not only recording but also fusion of the various recordings from multiple recordings, but has been shown for large collection efforts such as in pNEUMA^20^. Fourth, due to the single-laned track and the absence of crossing and overtaking manoeuvrers, vehicle tracking was simple and tracking ID switches could be avoided. In more complex, real-world setups, more elaborate tracking strategies and algorithms (e.g. DeepSORT^61^) might be necessary. Besides, future works might consider not only applying models trained on DOTA, but also on other datasets to increase generalizability.

**Pipeline robustness.** Previous studies indicated the potential of OBB for more robust object detection due to less occlusion and lower variance of backgrounds. Moreover, a previously observed challenge was environmental variability (e.g. low or uneven illumination, partial shading, inclined views) for the precision of trajectory extraction methods^26,27^. The simple conditions of this experiment and the consistency in lightning due to stable weather conditions do not allow to draw conclusions on the robustness of OBB detection with higher varying backgrounds (such as urban intersections or other real-world roads) and with varying lightning conditions. However, the findings of this study encourage to continue research on the benefits of OBB object detection for trajectory extraction. Future works might delve into exploring the benefits and robustness-gains of OBB at specific, urban intersection datasets such as^25^, or dedicated further experiments to analyse the impact of adverse lighting and weather conditions on trajectory accuracy.

**Run-time analysis and real-time applicability.** The proposed pipeline could be seamlessly incorporated to live-traffic monitoring applications. The computationally most expensive aspect of the pipeline is the object detection step, which due to the recent developments in computer vision and edge computing, already accelerated and became more accessible even in on-device applications. All other steps of the pipeline take a fraction of that time (ca. 5%). The processing of this study’s video material (40 minutes) took 10 minutes (excluding the object annotation) on a common, state-of-the art laptop (32GB RAM, Intel i7-1260P, 21000 MHz, 12 core processor). While here we used a high frame-rate of 25 FPS, real-time applications on highways or urban intersections might suffice with lower frame rates of around 10 FPS already, better suiting the computational abilities of available devices. The optimal choice of the frame-rate however should be determined at the speed of vehicles to be observed, to ensure that matching and tracking algorithms still work properly.

## Data Availability

The datasets generated and/or analysed during the current study are available in the repositories, as outlined in the following. The source code to reproduce the findings of this study and run the computational pipeline for another dataset can be found on GitHub: https://github.com/DerKevinRiehl/trajectory_analysis. The data (original video files, annotations, and trajectories) can be found on Zenodo: https://zenodo.org/records/15124431.
